# A Case of BRASH Syndrome Required Two Hospitalizations in a Short Period

**DOI:** 10.7759/cureus.72731

**Published:** 2024-10-30

**Authors:** Koichi Zokumasu, Yuki Natori, Masaki Kawakami

**Affiliations:** 1 Emergency Medicine, Kanto Central Hospital, Setagaya-Ku, JPN

**Keywords:** antiepileptic agent, bradycardia, brash syndrome, elderly individuals, emergency medicine

## Abstract

BRASH syndrome, characterized by bradycardia, renal failure, atrioventricular (AV) nodal blockade, shock, and hyperkalemia, is a recently identified syndrome typically caused by the interplay of electrolyte imbalances and medications such as beta-blockers and calcium channel blockers. This report presents the case of a 79-year-old woman with a history of epilepsy and hypertension, managed with carbamazepine, lamotrigine, and antihypertensive medications. She developed BRASH syndrome following reduced fluid intake and worsening renal function. Despite treatment for hyperkalemia and dehydration, she experienced two separate episodes of severe bradycardia, both requiring hospitalization. The second episode was more severe, leading to the placement of a permanent pacemaker. Interestingly, the patient did not exhibit hypotension, which is often associated with BRASH syndrome, highlighting the variability in its presentation. Furthermore, the involvement of antiepileptic drugs like carbamazepine and lamotrigine in this case suggests that BRASH syndrome may not be limited to the effects of cardiovascular medications. This case underscores the importance of early recognition and comprehensive management of BRASH syndrome, particularly in patients taking multiple medications. It also emphasizes the need for further research into the pathophysiology, treatment, and long-term prognosis of this emerging syndrome.

## Introduction

BRASH syndrome, characterized by bradycardia, renal failure, and hyperkalemia, emerges in patients treated with medications that can inhibit atrioventricular (AV) conduction, such as calcium channel blockers and beta-blockers. The syndrome can develop when an initiating factor worsens renal function leading to increased drug levels in blood, or when the dosage of these drugs is increased. These changes, coupled with renal dysfunction-induced hyperkalemia, lead to bradycardia due to the synergistic effects of the medications and hyperkalemia. This syndrome is marked by severe bradycardia precipitated by more subtle changes compared to those caused by AV nodal-blocking drugs or hyperkalemia alone. Furthermore, the resultant bradycardia can reduce renal blood flow, lead to further deterioration of renal function, and trigger a fatal negative spiral [1]. Angiotensin receptor blockers (ARB) are known to exacerbate hyperkalemia, acting as a modifier in this spiral. Another fundamental aspect of BRASH syndrome is the reversibility of the severe bradycardia it induces, contingent upon timely and appropriate intervention. There are documented instances where severe bradycardia has been effectively managed with solely non-invasive treatment approaches [2-4]. Furthermore, there are some reports detailing cases where patients, initially requiring temporary pacing, were successfully weaned off without the subsequent permanent pacemaker implantation [5-7].

Since its proposal by Farkas in 2016, many cases of BRASH syndrome have been reported, and understanding of the condition is gradually accumulating. However, the syndrome is still often under-recognized.

## Case presentation

A 79-year-old woman was brought to the emergency department by ambulance due to dizziness and vomiting. Upon waking on a hot early summer morning, she experienced positional vertigo that improved with rest but worsened with movement. She had occasionally felt dizzy before and chose to wait and see if the symptoms would resolve on their own, but as her nausea intensified and she vomited twice, she called an ambulance.

She had a history of temporal lobe epilepsy, treated with carbamazepine 300 mg twice daily, and lamotrigine 100 mg once daily. Additionally, she suffers from hypertension, managed with nifedipine 40 mg twice daily and azilsartan. Two weeks before her emergency transport, during a routine outpatient visit, her dose of azilsartan was increased from 20 mg to 40 mg daily. She had been adhering to her medication regimen without any overuse or missed doses.

After undergoing bilateral knee arthroplasty at another hospital, she gradually became reluctant to move and even tried to avoid going to the bathroom as much as possible. To reduce the frequency of bathroom visits, she intentionally limited her fluid intake.

**Figure 1:**
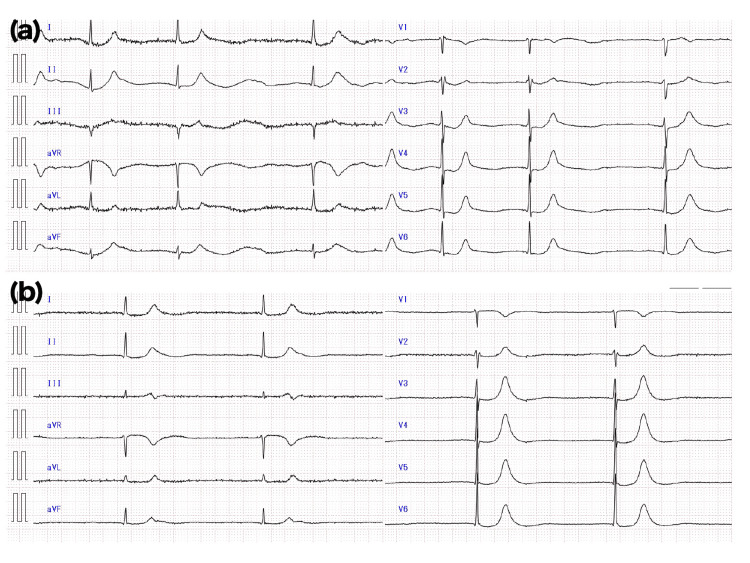
ECG at the emergency department ECG at the time of the first admission (a) and second admission (b) showing bradycardia. ECG, electrocardiogram

Upon arrival, her blood pressure was 198/83 mmHg, and her body temperature was 37.1 °C. There were no obvious signs of nystagmus, no limb paralysis, and no speech disturbances. An electrocardiogram (ECG) showed a junctional rhythm with a heart rate in the 30s (Figure 1a). Laboratory results revealed a potassium level of 5.5 mmol/L (reference range: 3.3-4.8 mEq/L), blood urea nitrogen (BUN) of 46 mg/dL (reference range: 8-20 mg/dL), and creatinine of 1.87 mg/dL (reference range: 0.4-0.8 mg/dL). 

To stabilize the membrane potential of cardiomyocytes, intravenous calcium gluconate was administered. Additionally, a mixture of regular insulin in glucose solution was given to promote an intracellular shift of potassium. Following this treatment, her heart rate improved to 40-50 beats per minute (bpm). Her symptoms of dizziness also gradually improved.

She was admitted for observation and further management. During her hospital stay, she received oral polystyrene sulfonate for continued hyperkalemia management. Serial blood tests showed progressive improvement in her potassium levels, reaching 3.9 mmol/L by day seven, at which point the medication was discontinued. Renal function also improved, with creatinine levels reaching 0.91 mg/dL. At the time of discharge, her azilsartan dosage was reduced from 40 mg to 20 mg daily, considering the possibility that it may have contributed to the pathogenesis.　

One month later, she was again transported to the emergency department due to dizziness and nausea. As in her previous emergency admission, her serum potassium was elevated at 5.2 mmol/L, and creatinine had increased to 2.61 mg/dL. An ECG showed bradycardia with a heart rate of 31 bpm (Figure 1b). Calcium gluconate and insulin with glucose were administered, and she was admitted. During the second hospitalization, a more detailed examination was conducted. Thyroid function tests showed TSH of 2.45 μIU/mL (reference range: 0.35-4.94 μIU/mL), free T3 of 1.89 pg/mL (reference range: 1.88-3.18 pg/mL), and free T4 of 0.79 ng/dL (reference range: 0.70-1.48 ng/dL), revealing no significant abnormalities. The blood concentration of carbamazepine was 9.0 μg/mL (reference range: 4.0-12.0 μg/mL), which was within the normal range. The blood concentration of Iamotrigine was 2.41 μg/mL (reference range: 2.50-15.00 μg/mL), rather low.

This time, the bradycardia was more severe than during the previous admission, with the heart rate dropping into the 20s on ECG monitoring after admission, along with frequent pauses lasting several seconds. After consultation with the cardiology team, the decision was made to temporarily place a pacemaker. Subsequently, in collaboration with the neurology department, although the possibility that the medications for temporal lobe epilepsy were contributing to the condition could not be completely ruled out, reducing or discontinuing these medications was deemed challenging due to their role in seizure control. Taking these factors into consideration, and considering the patient's preference, the decision was made to implant a permanent pacemaker. After pacemaker placement, the patient was discharged home.

## Discussion

BRASH syndrome (bradycardia, renal failure, AV nodal blockade, shock, and hyperkalemia) is characterized by the interplay of renal dysfunction, hyperkalemia, and AV nodal blockade leading to severe bradycardia. This bradycardia can lead to a decrease in blood pressure, reducing renal perfusion pressure, thereby impairing renal function. This reduction in renal function can lead to increased blood concentrations of drugs that block AV conduction. Without interventions to break this cycle, improvement in the condition is unlikely [8]. While case reports are gradually accumulating, many aspects of its pathophysiology, treatment, and prognosis remain unclear. 

One reason that complicates the diagnosis of BRASH syndrome is that many patients exhibit only mildly elevated serum potassium levels, much lower than those typically associated with arrhythmias caused by hyperkalemia alone [3,9-12]. This subtlety in electrolyte imbalance can delay recognition and intervention, posing continued risks to patients.

Additionally, the management of BRASH syndrome is hindered by the limitations of standard Advanced Cardiac Life Support (ACLS) protocols. Treatments like atropine, usually effective for bradyarrhythmia, do not typically improve outcomes in BRASH syndrome patients [5,11].

Furthermore, while many reports of BRASH syndrome involve patients with pre-existing chronic kidney disease (CKD), the patient in this case did not have prior CKD [4,5,7,9,10,12-14]. The deterioration in renal function observed here was likely triggered by her intentionally reduced fluid intake to lessen the need for bathroom visits due to bilateral knee osteoarthritis pain, compounded by the high temperatures of early summer. In fact, other reports suggest that increases in summer temperatures can precipitate BRASH syndrome, highlighting how seemingly minor triggers can initiate a cascade leading to severe bradycardia [15]. This underscores the potentially precarious nature of BRASH syndrome, where a slight trigger can set off a critical spiral into bradycardia. 

Reports of BRASH syndrome frequently identify beta-blockers or calcium channel blockers as the suspected culprit, and ARB has also been implicated as a contributing factor [16]. In this case, the patient was taking a calcium channel blocker. Additionally, the patient was taking antiepileptic medication carbamazepine and lamotrigine. There are conflicting reports regarding the association between carbamazepine and bradyarrhythmia, with some studies suggesting it as a potential side effect and others denying such a link [17,18]. Moreover, lamotrigine has been flagged by the FDA as having the potential to suppress cardiac conduction, leading to revisions in its labeling [19]. This suggests that antiepileptic medication could have also played a role in this patient’s clinical course. Thus, beyond the commonly implicated beta-blockers, calcium channel blockers, and ARBs, other medications may contribute to the pathophysiology of BRASH syndrome. While further research is required in this area, clinicians should maintain a thorough understanding of all medications a patient is taking to ensure comprehensive management.

In BRASH syndrome, as the name suggests, it typically involves a decrease in blood pressure due to bradycardia. However, in this case, no hypotension was observed. There have also been other reports where no decrease in blood pressure was noted or cases where the blood pressure was initially elevated and later decreased [9,10]. Therefore, the patient's blood pressure, whether high or low, may simply reflect the timing of the measurement, and the absence of hypotension should not be used as a reason to rule out BRASH syndrome.

The BRASH syndrome is still an emerging concept with no long-term outcome reports yet available. In our emergency department, we have encountered three cases of BRASH syndrome in the past year; all initially responded to therapeutic interventions, but all cases progressed to irreversible bradycardia, ultimately requiring pacemaker implantation, suggesting that while immediate management was effective, the long-term prognosis for BRASH syndrome may not be as favorable. This highlights an urgent need for further research into this condition. Finally, the case reports on BRASH syndrome discussed thus far are summarized in Table 1.

**Table 1:** Reported cases of BRASH syndrome CHF: chronic heart failure, CKD: chronic kidney disease, HTN: hypertension, T1DM: type 1 diabetes mellitus, T2DM: type 2 diabetes mellitus, DM: diabetes mellitus, NASH: nonalcoholic steatohepatitis cirrhosis, AF: atrial fibrillation, GI: glucose and insulin, AVB: atrioventricular block, RBBB: right bundle branch block

Age	ECG before onset	Major comorbidity	Usual medication	Pulse rate (bpm)	Blood pressure (mmHg)	Potassium (mEq/L)	Creatinine (mg/dL)	Treatments	Reference
62 F	Not provided	CKD, CHF, HTN, hyperlipidemia, T2DM	Metolazone, bumetanide, carvedilol	31	63/32	8.0	4.06	Dopamine, atropine, GI, calcium gluconate, polystyrene sulfonate	Srivastava 2020 [5]
76M	Not provided	Psoriasis, CKD, HTN	Amlodipine, atenolol	26	80/40	7.3	3.7	Atropine, GI, calcium gluconate, epinephrin, temporary pacemaker	Bailuni 2022 [7]
77M	AF	CHF, AF, HTN, T2DM	Verapamil, lisinopril, furosemide	30	90/40	5.6	3.4	Atropine, GI, calcium gluconate	Shah 2022 [3]
86 F	Not provided	CHF	Metoprolol, lisinopril	30	Not provided	6.7	2.8	Calcium gluconate, GI, furosemide, sodium zirconium cyclosilicate	Shah 2022 [3]
55F	Not provided	CKD, HTN, T2DM	Diltiazem, hydralazine, bumetanide, metolazone, bumetanide, atorvastatin, insulin	30	82/37	5.4	13.5	Calcium gluconate, GI, polystyrene sulfonate, atropine, dopamine, temporary pacemaker, hemodialysis	Arif 2020 [13]
74F	AF	CHF, AF, CKD, T2DM	Rivaroxaban, carvedilol, perindopril, urapidil, atorvastatin, amlodipine, eplerenone, furosemide, empagliflozin, insulin, potassium tablets, vitamins	31	170/100	5.8	5.58	Atropine, dopamine, calcium gluconate, furosemide, norepinephrine, dopamine	Sedlak 2023 [9]
66F	AF	CHF, aortic stenosis, AF, CKD	Apixaban, diltiazem, metoprolol, potassium chloride, furosemide	20	Not provided	8.1	2.21	Temporary pacemaker, epinephrine, calcium gluconate, GI	Roma 2024 [14]
89F	1st AVB RBBB	HTN, depression	Labetalol hydralazine, isosorbide dinitrate, trazodone, lexapro, amlodipine, clonidine	25	50/not provided	5.8	1.68	Atropine, normal saline	Aiwuyo 2022 [20]
43F	AF	AF, HTN, DM, NASH, bipolar disorder	Diltiaze, metoprolol tartrate	35	109/42	7.6	2.75	GI, calcium gluconate, temporary pacemaker	Grigorov 2020 [6]
67M	AF	AF, T1DM, sleep apnea, CKD	Apixaban, amlodipine, flecainide, simvastatin, hydrochlorothiazide, losinopril, metoprolol, insulin	24	142/66	5.6	2.12	GI, calcium gluconate	Saini 2023 [10]

## Conclusions

This case underscores the importance of considering BRASH syndrome in elderly patients presenting with bradycardia, particularly those on medications affecting renal function and cardiac conduction. Early recognition and appropriate management are crucial for favorable outcomes, and further research is needed to elucidate the full spectrum of clinical presentations and underlying mechanisms of BRASH syndrome.

Additionally, the possibility of drug interactions, particularly with medications like carbamazepine or lamotrigine, should be kept in mind when assessing patients with complex medical histories. This case contributes to the growing body of evidence on BRASH syndrome and reinforces the need for comprehensive evaluation and management in similar clinical scenarios.
